# Avoidance of general anesthesia for circumcision in infants under 6 months of age using a modified Plastibell technique

**DOI:** 10.1007/s00383-019-04452-x

**Published:** 2019-02-14

**Authors:** Thanh Tam Nguyen, Elizabeth Kraft, Ziyad Nasrawi, Minal Joshi, Demetri Merianos

**Affiliations:** Department of Surgery, New York Presbyterian Brooklyn Methodist Hospital, 506 6th street, Brooklyn, NY 11215 USA

**Keywords:** Modified Plastibell technique, Circumcision, Local anesthesia, Infants, Risks of general anesthesia.

## Abstract

**Purpose:**

There is currently no gold standard for the type of analgesia or preferred circumcision technique in infants requiring circumcision after 1 month of age. Our study presents a modified Plastibell circumcision technique, which offers excellent surgical outcomes, and can be performed under local anesthesia until 6 months of age, thereby avoiding the risks of general anesthesia in delayed circumcision.

**Methods:**

This is a retrospective case series of 508 consecutive male infants between 1 and 6 months of age, from one institution, who all underwent circumcision under local anesthesia, performed by the same pediatric surgeon, from 2013 to 2018. The study parameters included postoperative complications such as re-operation for control of hemorrhage, wound infection, circumcision revision, and urethral meatotomy.

**Results:**

There were no re-operations for control of hemorrhage, no wound infections, and no circumcision revisions. One patient developed urethral meatal stenosis requiring urethral meatotomy.

**Conclusion:**

Our modified Plastibell circumcision technique under local anesthesia is a safe and reproducible alternative for infants between 1 and 6 months of age, whose parents desire circumcision and wish to avoid general anesthesia.

## Introduction

Approximately 1.5 million babies under the age of 1 undergo general anesthesia each year for surgical procedures in the US [1, 2]. While the medical benefits of circumcision are still debated within the medical literature, circumcision remains one of the most common surgeries performed in infants, often for cultural or religious reasons [3]. While practice patterns vary greatly around the world, within the New York metropolitan area, the vast majority of circumcisions are performed by pediatric and obstetric providers in the newborn nursery under local anesthesia, but most pediatric and obstetric providers do not offer circumcision after 1 month of age, or for infants over 10 pounds. Conversely, most pediatric general surgeons and pediatric urologists do not perform circumcisions without general anesthesia, and therefore, do not offer circumcision before 6 months of age, presumably to mitigate anesthetic risks.

The risks of general anesthesia in infants are many, and have been studied extensively over the past decade. These risks include apnea and bradycardia in children under 6 months of age, laryngospasm in children under 1 year of age, and the concern for long-term effects on brain development in children under 3 years of age [4–6]. There is currently no gold standard for the type of analgesia or preferred circumcision technique in infants requiring circumcision after 1 month of age. This study’s aim is to present a modified Plastibell technique, with a favorable intra-operative and post-operative risk profile, that can be performed under local anesthesia until 6 months of age, thereby avoiding the risks of general anesthesia.

## Methods

### Patient selection and study design

The subjects included in this retrospective case series were 508 consecutive male infants between 1 and 6 months of age, from one institution, all undergoing circumcision under local anesthesia between January 2013 and December 2018. The mean patient age was 3.34 months, with a variance of 0.64 months. The predominant pre-operative diagnosis was congenital phimosis, with the vast majority of circumcisions being performed for cultural or religious reasons, and fewer than 10% being performed for acquired phimosis, balanitis, or recurrent urinary tract infections. The procedures were performed in an operating room within the ambulatory surgery center of New York Presbyterian Brooklyn Methodist Hospital. The only known co-morbidity was premature delivery, which involved 8% of patients. Institutional IRB approval was obtained: ID 1135503-1. All cases were performed by the same pediatric general surgeon. The study parameters include postoperative complications such as re-operation for control of hemorrhage, wound infection, circumcision revision, or urethral meatotomy.

### Pre-surgical preparation

All parents are instructed to feed the infant up until 5–10 min prior to surgery, to allow for belching prior to surgery. The room is warmed to 72 degrees, and the infant is placed in the supine position on a warming device (infant bear hugger), with oxygenation monitoring via pulse oximetry on the foot, which records both heart rate and oxygen saturation. Intravenous access is not necessary for this procedure, and is, therefore, not obtained. A nurse stands at the head of the bed, and gently holds the patient’s knees in place, to prevent contamination of the sterile field. The operating room table is placed in a slight reverse Trendelenburg position, to minimize reflux and spitting up, given the short NPO times. The surgical site is prepped with betadine, and sterile drapes are placed, with care not to cover the infant’s face, and not to shine operating room lights into the infant’s eyes. The Bovie pad is placed on the infant’s lower back.

### Anesthesia

The procedure is done under local anesthesia via penile block which involves a dorsal penile nerve block (DPNB) and a penile ring block. A 40 mg/kg acetaminophen rectal suppository is administered in all infants before local anesthesia, and the penile block is injected using a 25-gauge needle. A 50/50 mixture of 1% Lidocaine and 0.25% Bupivacaine is used for both dorsal penile nerve block (infiltration just inferior to the pubic symphysis at 11 o’clock and 1 o’clock), as well as penile ring block (superficial injection at the base of the penis circumferentially), for all infants, with a total injected volume of 0.75–1.0 cc per kg. We prefer to use 80% of the injected volume on the ring block, as we have found it to be the more effective of the two. Three minutes are allowed to pass, to allow the local anesthesia to take effect, and then a mosquito clamp is used to clamp the foreskin while observing the patient’s reaction and confirming complete analgesia prior to beginning the circumcision. If an infant cries for more than 3 min after the administration of the nerve block, oral sucrose is provided via a pacifier.

### Surgical technique

**Fig. 1:**
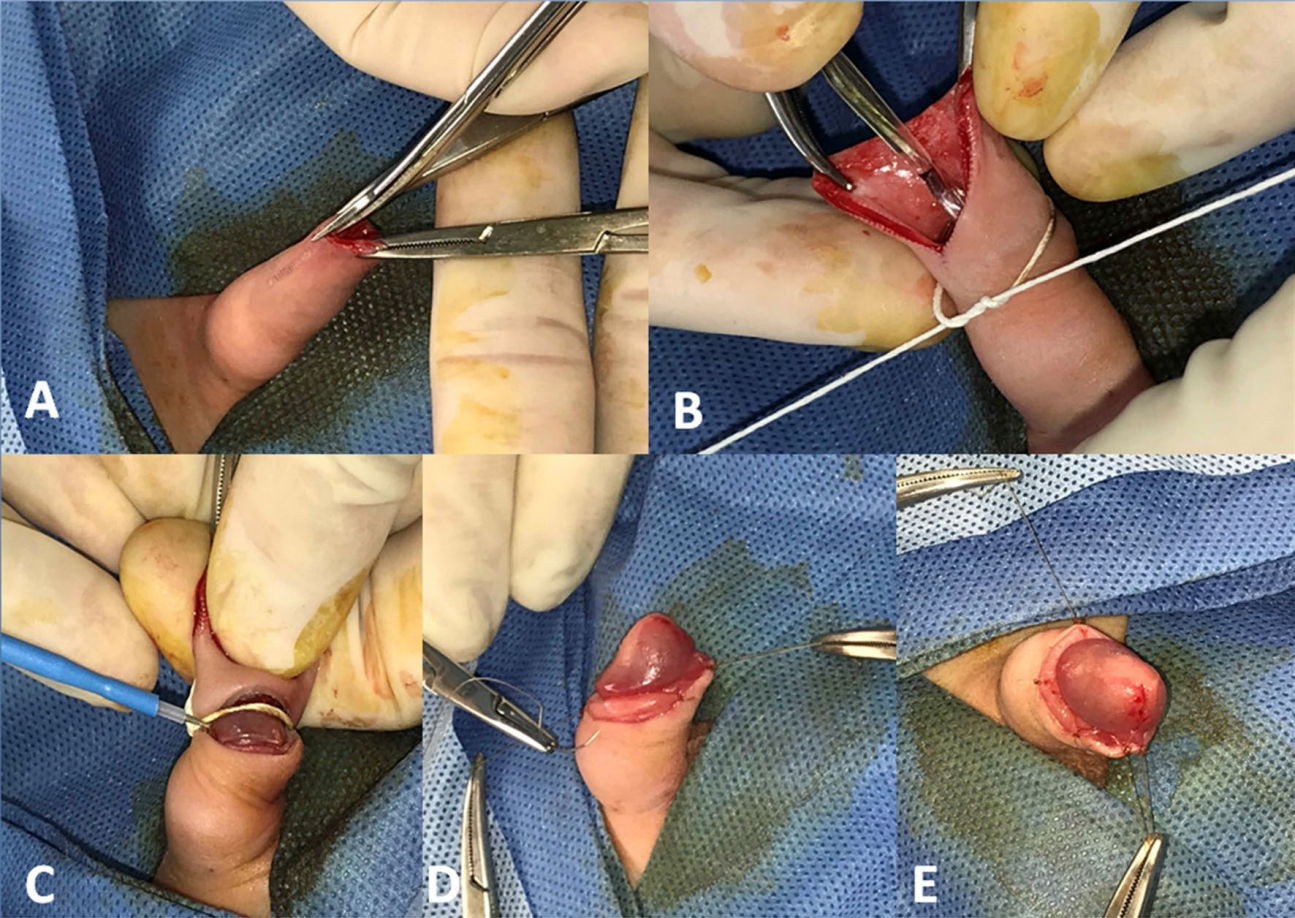
**a** Dorsal slit. **b** Plastibell placement. **c** Foreskin Division. **d** Dorsal and Ventral Stitches. **e** Complete circumcision

The foreskin is retracted and separated from the head of the penis, the smegma is removed, and the head of the penis is re-prepped with betadine. In cases where the frenulum of the penis appears to be foreshortened, a penile frenulotomy is performed with electrocautery. A straight clamp is used to clamp the foreskin in the region of the dorsal slit, for the purpose of hemostasis. The dorsal slit is then performed with a scissor (Fig. 1a). After selecting the appropriate Plastibell size, the Plastibell device is then placed over the glans of the penis, and the string of the device is tied down firmly within the groove (Fig. 1b).

Unlike the traditional Plastibell technique, where the foreskin would now be divided distal to the string, in our technique the foreskin is divided on the proximal side of the string using needle tip Bovie electrocautery. The Bovie generator mode is set to cutting, and the power set to 12 Watts (Fig. 1c). The Plastibell device, string, and foreskin are then discarded, and meticulous hemostasis is achieved with electrocautery, with the generator mode set to coagulation, and the power set to 12 Watts. Once complete hemostasis is confirmed, the skin–mucosal interface is re-approximated using simple, interrupted 5 –0 chromic sutures, with two stitches being placed ventrally, one stitch being placed dorsally, and 2–3 stitches being placed laterally on each side, for a total of 7–9 stitches (Fig. 1d and e). This circumcision procedure typically takes less than 20 min, and is performed with less than 3 cc of blood loss.

### Post-surgical instructions

After the procedure, bacitracin antibiotic ointment is applied, without any other surgical dressings, and a new diaper is placed. The patient is transported directly from the operating room to the arms of their parents in the waiting room, as no recovery room stay is required at our institution for cases under local anesthesia, without sedation. Parents are given additional bacitracin ointment and instructed to apply the bacitracin ointment during each diaper change for the next 2 days. Parents are told that they may give an additional dose of infant Tylenol 6 h after the procedure, if needed, for post-operative analgesia. Each infant is seen in the office in 2–3 weeks, for post-operative follow-up.

## Results

We performed 508 consecutive circumcisions under local anesthesia in children age 1–6 months, with a mean age of 3.34 months, and all patients were followed for at least 2 weeks post-operatively. We report no re-operations for control of hemorrhage, no wound infections, and no circumcision revisions. There were no adverse intraoperative events, and no procedures were delayed or aborted for patient discomfort or movement. There were no incidences of aspiration or intra-operative desaturation. One patient developed urethral meatal stenosis, requiring urethral meatotomy 6 months post-operatively.

## Discussion

It has been our experience that several infants leave the newborn nursery without having been circumcised, despite parental desire. Within the New York metropolitan area, this trend is increasing, as more and more obstetricians are no longer performing circumcisions. These patients often present to pediatric general surgeons or pediatric urologists, who then often delay circumcision until after 6 months of age and perform this procedure under general anesthesia. Although the risks of apnea and bradycardia are low at this age, the risk of laryngospasm is not insignificant. Additionally, on December 14, 2016, the US. Food and Drug Administration warned that “repeated or lengthy use of general anesthetic and sedation drugs during surgeries or procedures in children younger than 3 years…may affect the development of children’s brains.”[7].

We utilize a dorsal penile nerve block, a ring block, and a 40 mg/kg rectal loading dose of Tylenol, in addition to selective use of oral sucrose via a pacifier for the few infants who do not stop crying within 3 min after the injection of our penile block, which is the minimum period of time we wait for the block to take effect before starting the circumcision. Our choice of analgesia is based on an extensive examination of the literature regarding analgesic strategies for neonatal circumcision. Specifically, dorsal penile nerve block and ring block have both been shown to provide more effective analgesia than topical analgesic cream or oral sucrose in multiple studies [8–14]. Our technique avoids the risks of general anesthesia, without sacrificing patient comfort. The key to the technique is the injection of an adequate amount of local anesthetic, and then waiting at least 3 min for the block to take effect before performing the circumcision. We also test each patient by clamping the foreskin with a hemostat, and we do not proceed until we have confirmed analgesia via complete lack of response to the application of the hemostat. We typically inject 1 cc per kilogram of our 50:50 mixture of 1% lidocaine and 0.25% bupivacaine, with the majority being used on the ring block, and not the dorsal block.

Although the presence or absence of pain or crying was not recorded in our operative reports, and therefore, cannot be presented scientifically, we would like to anecdotally discuss the degree of pain experienced by our patients, both intra-operatively and post-operatively, utilizing this technique, as that is an important concern regarding the use of this technique in infants over 1 month of age. With regard to intra-operative pain, 100% of the infants do cry during the injection of local anesthesia, which takes approximately 5 s, but is no more painful than a routine vaccination. The younger infants (0–3 months) typically settle down within seconds of when the injection has concluded, while the older infants (4–6 months) can take 3–5 min to settle down. We have anecdotally found that approximately half of our infants are able to sleep through the circumcision itself, while approximately 5% of them will cry throughout the procedure. In addition to injecting an adequate volume of local anesthesia, and waiting at least 3 min for it to take effect, we have found that the most important factors in terms of preventing the baby from crying during the procedure, are making sure that the infant has been fed prior to the procedure, while allowing 5–10 min for belching the baby after feeding, and maintaining a warm room temperature (ideally 72 degrees). The importance of these measures cannot be overstated, as we have seen the percentage of crying babies gradually decrease over the years, as we have become more meticulous about following these guidelines.

Furthermore, every parent was asked about post-operative pain duration and need for Tylenol during the follow-up office visit, and approximately 50% of parents reported no post-operative pain or need for Tylenol, while approximately 50% reported 24 h of post-operative fussiness, particularly during diaper changes, which was controlled with Tylenol.

There are several acceptable circumcision techniques, with the three most popular techniques being the conventional Plastibell technique, the Gomco clamp technique, and the Mogen clamp technique. All of these techniques can be performed with favorable risk profiles in experienced hands, however, we do feel that our approach offers certain advantages. Specifically, we believe that our modification to the traditional Plastibell technique allows us to obtain more complete hemostasis than the traditional clamp techniques, as we have the opportunity to individually cauterize any bleeding vessels prior to placement of our stitches. This is further supported by the fact that we experienced no re-operations for bleeding in over 500 cases, compared to 0.5–1% with most other techniques [15], and a 1.1% postoperative bleeding risk in one cross-sectional study where the conventional Plastibell technique was performed in over 2000 infants similar in age to the patients in our study [16]. Another historical cohort that we can utilize as a control group for the purpose of comparison involves a group of 4500 infants in Egypt who underwent circumcision with a conventional Plastibell device, with a reported hemorrhage risk of 1.5% [17].

Lastly, our approach minimizes the need for circumcision revision for excess foreskin, because we have the opportunity to remove additional foreskin after removal of the Plastibell, if necessary. Although we did not record the need to trim additional foreskin in our dictated operative reports, anecdotally we have found this to be necessary in up to 5% of cases, and this additional trimming is not an option with the traditional Plastibell technique, potentially leading to the more frequent need for circumcision revision in those cases. The traditional Plastibell technique can also result in incomplete separation or slippage of the ring, leading to penile injury, tissue necrosis and disfigurement [18–20]. Lastly, post-operative care with our technique is minimal for parents, as we allow them to utilize baby wipes as soon as the day of surgery.

While we recognize that our study is limited by a relatively small sample size, very short term follow-up and the fact that its single surgeon design precludes us from drawing any conclusions about its widespread applicability, we feel that we have demonstrated that our technique is no more complex than traditional circumcision techniques, and provides comparable results, without the need for general anesthesia. As previously mentioned, a significant limitation of this study is the short follow-up period, and the possibility of patients presenting to other providers for issues that may arise after their post-operative office visit. However, we feel that this technique is highly reproducible, with low complication rates, and aesthetically favorable surgical outcomes.

## Conclusion

This modified Plastibell technique under local anesthesia is a safe and effective option for children between 1 and 6 months of age. It avoids the risks of general anesthesia, is comfortable for the infant, and is associated with lower risks of bleeding when compared to traditional clamp techniques.
